# Different distances between central venous catheter tips can affect antibiotic clearance during continuous renal replacement therapy

**DOI:** 10.1186/s40635-024-00635-6

**Published:** 2024-06-24

**Authors:** Anna Bandert, Miklós Lipcsey, Robert Frithiof, Anders Larsson, David Smekal

**Affiliations:** 1 Department of Surgical Sciences, Anaesthesiology and Intensive Care, Uppsala University, Uppsala, Sweden; 2 Centre for Research and Development, Uppsala University,Region Gävleborg, Gävle, Sweden; 3 Department of Anaesthesiology and Intensive Care, Gävle Hospital, Lasarettvägen 1, 80324 Gävle, Sweden; 4 Department of Surgical Sciences, Hedenstierna Laboratory, Uppsala University, Uppsala, Sweden; 5 Department of Medical Sciences, Clinical Chemistry, Uppsala University, Uppsala, Sweden

**Keywords:** Central venous catheter, Intensive care, Continuous renal replacement therapy, Antibiotic concentration, Dialysis, Acute kidney injury, Renal replacement therapy, Clearance

## Abstract

**Background:**

The aim of this experimental study was to elucidate whether different distances between central venous catheter tips can affect drug clearance during continuous renal replacement therapy (CRRT). Central venous catheters (CVCs) are widely used in intensive care patients for drug infusion. If a patient receives CRRT, a second central dialysis catheter (CDC) is required. Where to insert CVCs is directed by guidelines, but recommendations regarding how to place multiple catheters are scarce. There are indications that a drug infused in a CVC with the tip close to the tip of the CDC, could be directly aspirated into the dialysis machine, with a risk of increased clearance. However, studies on whether clearance is affected by different CVC and CDC tip positions, when the two catheters are in the same vessel, are few.

**Methods:**

In this model with 18 piglets, gentamicin (GM) and vancomycin (VM) were infused through a CVC during CRRT. The CVC tip was placed in different positions in relation to the CDC tip from caudal, i.e., proximal to the heart, to cranial, i.e., distal to the heart. Serum and dialysate concentrations were sampled after approximately 30 min of CRRT at four different positions: when the CVC tip was 2 cm caudally (+ 2), at the same level (0), and at 2 (− 2) and 4 (− 4) cm cranially of the tip of the CDC. Clearance was calculated. A mixed linear model was performed, and level of significance was set to *p* < 0.05.

**Results:**

Clearance of GM had median values at + 2 cm, 0 cm, − 2 cm and − 4 cm of 17.3 (5.2), 18.6 (7.4), 20.0 (16.2) and 26.2 (12.2) ml/min, respectively (*p* = 0.04). Clearance of VM had median values at + 2 cm, 0 cm, − 2 cm and − 4 cm of 16.2 (4.5), 14.7 (4.9), 19.0 (10.2) and 21.2 (11.4) ml/min, respectively (*p* = 0.02).

**Conclusions:**

The distance between CVC and CDC tips can affect drug clearance during CRRT. A cranial versus a caudal tip position of the CVC in relation to the tip of the CDC led to the highest clearance.

## Background

Central venous catheters (CVCs) are widely used in critically ill patients. Between 60% and 90% of patients in the intensive care unit (ICU) are reported to have an indwelling CVC [1]. The most common indication for a CVC is infusion of vasoactive drugs, but they are also used for infusion of other life-supporting drugs and for haemodynamic monitoring [1]. Approximately 4–13% of all ICU patients will receive renal replacement therapy (RRT), most commonly due to sepsis-induced acute kidney injury [2–4]. If RRT is required, a central venous dialysis catheter (CDC) is inserted. Where these different catheters are inserted depends on patient habitus, previous central lines, bleeding risks, etc*.* [1, 5] There are guidelines on how and where to put CVC and CDC catheters, but in the case of several catheters in the same patient, the choice of placement is at the discretion of the treating physician [6, 7].

The tips of these two catheters are often positioned in the same vein, the superior or inferior caval vein (SCV or ICV). If the tip of the CVC is close to the tip of the CDC, there is a risk of direct aspiration of drugs into the RRT circuit and immediate elimination, leading to a lower blood concentration of drug than intended [8, 9]. An occurrence similar to direct aspiration is recirculation during extra corporeal membrane oxygenation (ECMO) and haemodialysis [10–13]. Recirculation leads to a reduction of oxygenation in ECMO and a lowered clearance in haemodialysis, whereas in the case of direct aspiration during RRT, there is a risk of a higher clearance of drugs. There are case reports where direct aspiration from the CVC to CDC during CRRT is suspected [14, 15] and Kam et al. also demonstrated the possibility of direct aspiration in a bench model. Kam et al. also showed differences in the amount of direct aspiration depending on different distances between the tips of the CVC and the CDC [16]. Vicka et al. showed in an in vitro model that different catheter tip positions, in the same vessel, result in different risks of direct aspiration [17]. In previous studies, we have shown that antibiotic clearance and noradrenaline dose can be affected by catheter tip vicinity with higher clearance and higher noradrenaline dose if the tips are in the same caval vein, compared to if the tips are in separate vessels [8, 9].

One possible way to avoid direct aspiration might be to position the CVC and CDC in different vessels. However, this is not always feasible in an ICU patient. We, therefore, wished to study whether different distances between the two catheter tips in the same vessel can influence clearance during continuous RRT.

## Methods

### Animals and ethics

The study was performed on eighteen 8–10-week-old Norwegian Landrace piglets, weight 27 ± 4 kg. The experiments were performed at Hedenstierna Laboratory, Uppsala University, Sweden.

The experiment was approved by Uppsala University’s Animal Ethics Committee (C 155/14 and 5.8.18-08592/2019). All animals were handled according to the guidelines of the Swedish Board of Agriculture and the European Convention on Animal Care. The study was reported in adherence to the Animal Research: Reporting of In Vivo Experiments (ARRIVE 2.0) guidelines [18, 19].

### Anaesthesia and preparation

Anaesthesia was induced by an intramuscular injection of a mixture of tiletamine–zolazepam 6 mg/kg and xylazine 2.2 mg/kg. Thereafter, anaesthesia was intravenously maintained by a continuous infusion of sodium pentobarbital 8 mg/kg/h and morphine 0.26 mg/kg/h dissolved in 2.5% glucose. Rocuronium bromide 1–3 mg/kg/h was infused continuously. This dose is to prevent shivering and not to give a total muscular block. The piglets were able to trigger in the ventilator or make small movements. If signs of insufficient anaesthesia were observed by movement, increasing heart rate or blood pressure, anaesthesia was immediately deepened by a bolus injection of ketamine and with a subsequent increase of infusion rate. Ringer’s acetate solution was administered at 1 ml/kg/h, resulting in a total fluid administration rate of approximately 10 ml/kg/h.

A tracheotomy was performed. The animals were mechanically ventilated throughout the experiment (Servo-I, Maquet, Stockholm, Sweden) with initial settings of respiratory rate 25/min, inspired oxygen fraction 0.3 and peak end expiratory pressure (PEEP) was set to 5 cmH_2_O. Tidal volume was approximately 8–10 ml/kg which at baseline yielded an arterial partial pressure of carbon dioxide (p_a_CO_2_) of 4.5–5.5 kPa. Respiratory rate was adjusted to keep p_a_CO_2_ at baseline level.

A central venous catheter (CVC) and a 13.5 Fr dialysis catheter (CDC) (Hemo-Cath SDL136E, MedCOMP, Harleysville, PA, USA) were inserted through the right external jugular vein (EJV) into the SCV. The tip is tapered with venous holes approximately 2 cm from the arterial holes.

A cervical artery was catheterized for pressure monitoring and blood sampling. A 7 Fr Swan-Ganz catheter was inserted in the pulmonary artery from the left EJV for monitoring. A urinary catheter was introduced via a bladder incision.

### Dialysis

The CRRT machine (MultiFiltrate, Fresenius Medical, Stockholm, Sweden) was prepared with a priming kit (multiFiltrate Ci-Ca® CVVHD 1000) with integrated polysulfone filter membrane (Ultraflux® AV 1000S) with a 1.8m^2^ surface area.

CRRT was started with continuous venovenous haemodialysis (CVVHD) settings and kept throughout the experiment: blood flow 60 ml/min (approximately 2.2 ml/kg/h), dialysate flow 1200 ml/h, citrate target 4 mmol/L, post-filter ionized calcium level 0.25–0.34 mmol/L, ultrafiltration 0 ml/min.

### Protocol

The pigs were anaesthetized as stated above. At the beginning of the preparation, an infusion of vancomycin, 4.55 mg/ml, was started in a peripheral vein at 9 mg/kg for 30 min and thereafter reduced to 3 mg/kg/h throughout the rest of the experiment. The vancomycin infusion rate after 30 min was 17 ± 2.6 ml/h. Gentamicin, 10 mg/ml, was infused with 3 mg/kg for 30 min and thereafter reduced to 1.5 mg/kg/h. The gentamicin infusion rate after 30 min was 3.8 ± 0.6 ml/h. The dose regimens were calculated to reach steady state. When the infusion rate was lowered, the infusions were moved to the CVC.

CRRT was started, followed by a 30-min stabilization period to ensure that the venous access and dialysis circuits were functioning adequately.

**Fig. 1:**
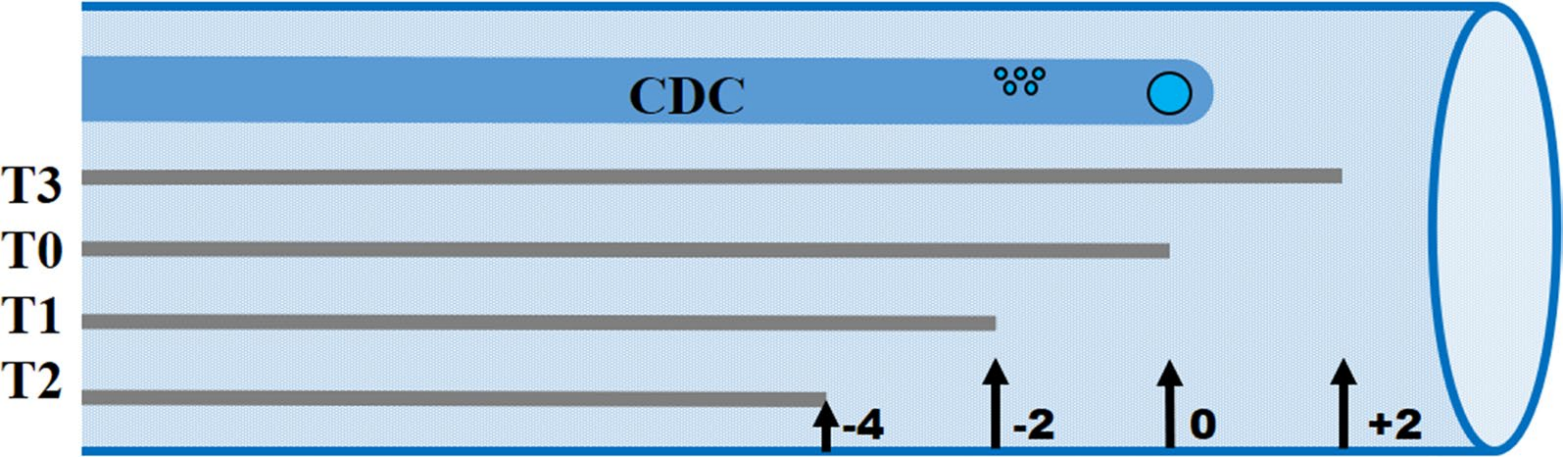
Central dialysis catheter (CDC) and central venous catheter (CVC) tip positions. The CDC has the outflow port, i.e., venous, at the tip of the catheter and inflow holes, i.e., arterial, at approximately 2 cm cranially from the tip. Each grey line represents the same catheter placed in four different positions. The CVC tip was placed in different positions in relation to the CDC tip from caudal, i.e., proximal to the heart, to cranial, i.e., distal to the heart at each position, marked with black arrows, antibiotic concentration samples were taken in blood and dialysate and dialysate clearance was calculated. Sampling started at 0 cm (T0) and the CVC was withdrawn cranially to − 2 cm (T1) and − 4 cm (T2). Thereafter, the CVC was placed caudally to the CDC at + 2 cm (T3)

The tips of the CVC and CDC were placed in close proximity to each other with the tip of the CDC 2 cm cranially from the atrio-caval junction. The position was confirmed using fluoroscopy. After 1 h and 20 min of antibiotics infusion (T0), concentration samples of gentamicin and vancomycin were taken from dialysate and blood via the arterial line. After blood sampling, the tip of the CVC was withdrawn 2 cm cranially and the new position was again confirmed using fluoroscopy. After approximately 30 min (T1), concentration samples of gentamicin and vancomycin were taken from blood and dialysate. The procedure was repeated with the CVC tip 4 cm cranially from the start position (T2) and, lastly, 2 cm caudally from the start position (T3) (Fig. 1).

If needed, noradrenaline (20 µg/ml) was given in the CVC with a target MAP of 60. At the end of the experiment, anaesthetic depth was controlled using surgical forceps. If there were signs of insufficient anaesthesia, the anaesthesia was increased. Thereafter, the piglets were euthanized by intravenous potassium chloride.

### Laboratory analyses

Plasma and dialysate were obtained for analysis of gentamicin and vancomycin concentrations at the start position, at − 2, − 4 and + 2 cm from start position (0 cm). Samples of blood gases were obtained at the start of CRRT as well as at the beginning and end of the experiment. Blood samples for antibiotic concentration, blood gases and point of care analyses were taken from the arterial line. Blood gas and point care analyses were performed on an ABL 800 blood gas analyser (Radiometer, Brønshøj, Denmark). Gentamicin and vancomycin were analysed on an Architect ci16200 (Abbott Laboratories, Abbott Park, IL, USA) with reagents (1P31) from the same manufacturer.

Clearance was calculated as follows:

$${\text{Clearance}}\, = \,({\text{dialysate}}\;{\text{concentration}} \times {\text{dialysate}}\;{\text{flow}})/{\text{plasma}}\;{\text{concentration}}.$$

### Statistical analysis methods

Data were analysed using Jamovi 1.6.15.0 (IBM Corporation, Armonk, NY, USA) and Statistica 14.1 (Cloud Software Group Inc, Palo Alto, CA, USA). Testing for normal distribution was performed using the Shapiro–Wilk test or histogram. Normally distributed data are presented as mean ± standard deviation (SD); data with a non-normal distribution are presented as median and interquartile range (IQR). Differences in timepoint characteristics were tested for using an independent Student’s *t* test or Mann–Whitney *U* test according to normality. A mixed linear model was used to test for differences between catheter positions with catheter distance and intercept as fixed effects and piglets as random effect. The level of significance was set at *p* < 0.05.

## Results

Of the initial 18 piglets, one was excluded due to technical problems with the antibiotic infusion and thereby a risk of incorrect concentrations. All other piglets completed the protocol. One piglet became transiently bradycardic when given a cold saline bolus for cardiac output measuring. Heart rate returned to normal after the end of saline infusion. Two piglets became tachycardic, which was interpreted as insufficient anaesthesia. These animals were given an extra bolus of ketamine following which the anaesthesia was deepened by elevating the infusion rate of continuous sedation. The mean time at each tip position was 30 ± 9 min.

Vital parameters and chemical analyses are shown in Table 1. Arterial oxygen saturation, arterial carbon dioxide pressure and arterial oxygen pressure were stable throughout the experiment.

**Table 1:** Vital parameters at different timepoints

	T0	T1	T2	T3
HR (bpm)	84 ± 14	82 ± 16	79 ± 16	82 ± 17
MAP (mmHg)	74 ± 13	75 ± 14	79 ± 12	80 ± 9
CO (L/min)	2.1 ± 0.7	2.2 ± 0.6	2.3 ± 0.5	2.4 ± 0.7
pH	7.47 ± 0.06	7.47 ± 0.06	7.47 ± 0.05	7.47 ± 0.05
P-Lactate (mmol/L)	1.9 ± 0.5	1.8 ± 0.5	1.75 ± 0.5	1.75 ± 0.5
Hb (g/L)	82 ± 4.4	81 ± 4.7	79 ± 4.9	78 ± 4.3

T0 represents when catheters are at 0 cm; T1 represents − 2 cm; T2 represents − 4 cm, and T3 represents + 2 cm. Mean time between timepoints was 30 ± 9 min

*HR* heart rate, *bpm* beats per minute, *MAP* mean arterial pressure, *mmHg* millimetres of mercury, *CO* cardiac output, *l/min* litres per minute, *P-lactate* plasma lactate, *mmol/L* millimoles per litre, *Hb* haemoglobin, *g/L* grams per litre

**Fig. 2:**
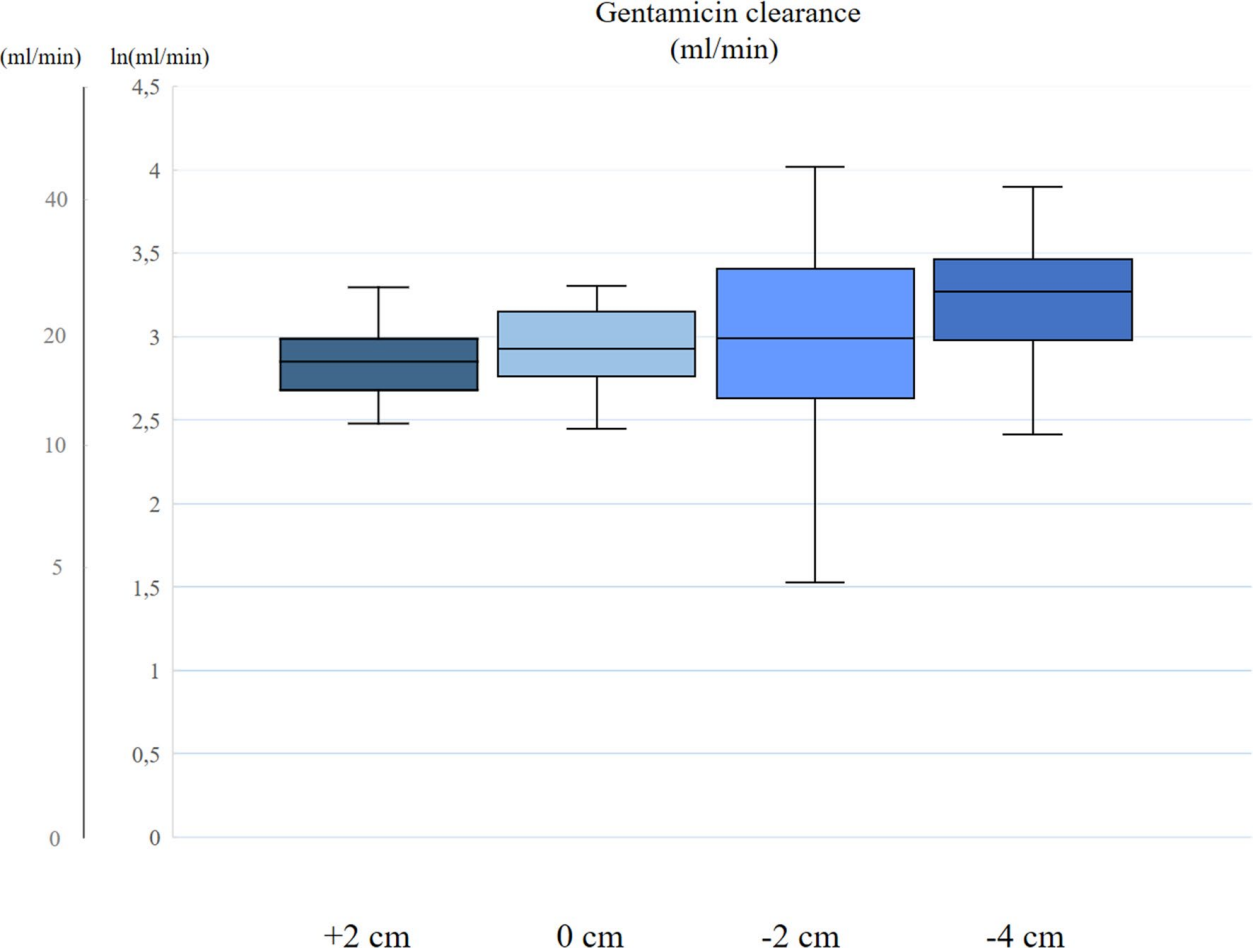
Clearance of gentamicin (GM). The boxplots show logarithmic dialysis clearance of GM at different distance between the CVC and the CDC tips. The line in the middle of the box indicate the median value, the whiskers represent the inter quartile range. The *y*-axis is in ml/min and ln ml/min. Clearance of GM had median values at + 2 cm, 0 cm, − 2 cm and − 4 cm of 17.3 (5.2), 18.6 (7.4), 20.0 (16.2) and 26.2 (12.2) ml/min, respectively (*p* = 0.04)

**Fig. 3:**
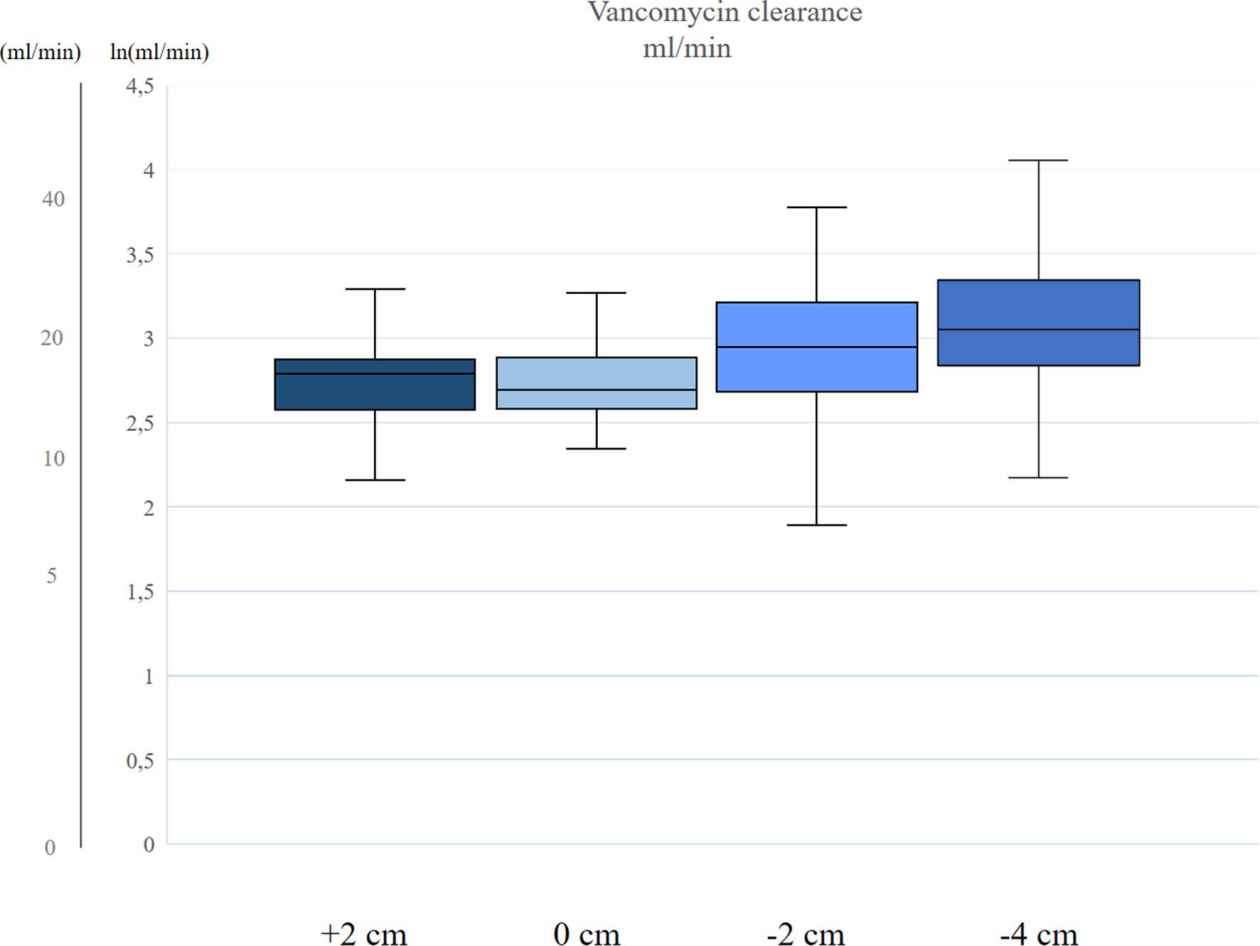
Clearance of vancomycin (VM). The boxplots show logarithmic dialysis clearance of VM at different distance between the CVC and the CDC tips. The line in the middle of the box indicate the median value, the whiskers represent the inter quartile range. The *y*-axis is in ml/min and ln ml/min. Clearance of VM had median values at + 2 cm, 0 cm, − 2 cm and − 4 cm of 16.2 (4.5), 14.7 (4.9), 19.0 (10.2) and 21.2 (11.4) ml/min, respectively (*p* = 0.02)

The clearance of GM and VM was different between the tip distances (see Figs. 2 and 3). Clearance of GM had median values at + 2 cm, 0 cm, − 2 cm and − 4 cm of 17.3 (5.2), 18.6 (7.4), 20.0 (16.2) and 26.2 (12.2) ml/min, respectively (*p* = 0.04). Clearance of VM had median values at + 2 cm, 0 cm, − 2 cm and − 4 cm of 16.2 (4.5), 14.7 (4.9), 19.0 (10.2) and 21.2 (11.4) ml/min, respectively (*p* = 0.02) (Table 2).

**Table 2:** Clearance and antibiotic concentrations at different tip distances

Position		+ 2 cm	0 cm	− 2 cm	− 4 cm	*p* value	*F* value
*Median (IQR)*							
Clearance (ml/min)	GM	17.3(5.2)	18.6(7.4)	20.0(16.2)	26.2(12.2)	0.04	2.9
	VM	16.2(4.5)	14.7(4.9)	19.0(10.2)	21.2(11.4)	0.02	3.6
Dialysate conc (mg/ml)	GM	3.2(2.1)	3.8(1.9)	5.43.4)	5.6(3.3)	0.003	5.4
	VM	7.9(3.2)	8.6(3.5)	10.1(5.0)	10.1(10)	0.03	3.3
Serum conc (mg/ml)	GM	4.99(3.2)	4.0(3.1)	5.1(2.9)	4.7(2.9)	0.9	0.2
	VM	11.0(6.1)	10.7(4.6)	10.6(2.0)	10.6(2.9)	0.4	0.9

Dialysate concentrations of GM and VM were also different between the various tip distances (Table 2). Serum concentrations of GM and VM did not differ between the various positions (Table 2).

CO did not affect GM clearance (*p* = 0.4, *F* 0.8) or VM clearance (*p*0 0.5, *F* 0.45).

## Discussion

The main result of this study is that antibiotic clearance can be affected by different distances between CVC and CDC tips when positioned in the same vessel during CRRT. A cranial versus a caudal tip position of the CVC in relation to the tip of the CDC led to the highest dialysis clearance. One plausible explanation for the results is direct aspiration of drugs from the CVC into the CDC.

Our results are consistent with results from our previous studies and also in vitro studies [8, 9, 16, 17].

The lowest clearance of antibiotics occurred when the CVC was 2 cm distal or at the same level as the CDC. This result is reasonable, since, in the absence of turbulent flow, the infused drug will follow the blood flow and will be carried away from the CDC. When the CVC tip was 2 cm proximal (− 2 cm) from the CDC tip, clearance was increased and was even greater at − 4 cm. However, a further increase in distance was not investigated and we did not answer the question of whether there is a proximal distance at which clearance decreases. In the in vitro study by Vicka et al., there was a peak in direct aspiration at − 4 cm [17].

The dialysate concentration was different between the catheter positions but not the serum concentration. A longer time between the position adjustments might have altered the serum concentration. CRRT in clinical use is often running for hours and days, which might have an effect on serum concentration of infused drugs.

In access recirculation in ECMO and RRT, a higher blood pump flow has been linked to an increased amount of recirculation [10–14]. One could speculate that a higher blood pump flow can also result in a higher amount of direct aspiration, but this was not investigated in this study. In analogy with the above reasoning, CO might as well be suspected to affect direct aspiration. In our experiment, CO did not affect GM clearance (*p* = 0.4, *F* 0.8) or VM clearance (*p*0 0.5, *F* 0.45). This is also in line with our previous results [8, 9].

In this animal model, two antibiotics were used as markers to investigate the phenomenon of direct aspiration. Clearance of gentamicin and vancomycin was 60–78% higher at − 4 cm compared to at + 2 cm and 0 cm. GM has a low level protein binding and VM a high level [20, 21]. Protein binding can affect adsorption in the dialysis filter membrane. Polysulfone membrane filters are shown to have very low adsorption of GM whereas VM is adsorbed rapidly but with no increase in adsorption after 30 min [22, 23]. Since the results show similar dialysis clearance of both antibiotics, it is unlikely that adsorption explain the results.

The priority of timely and correct antibiotic dose and regime has been well-described [24, 25]. Nevertheless, in the SMARRT trial, approximately 25% of the RRT patients failed to reach the target antimicrobial exposure using a standard dosing regimen [26].

Variations in antibiotic concentration in critically ill patients are multifactorial and even more so during RRT. Variation in concentration depends on factors such as volume of distribution, level of protein binding, molecular weight, hydrophilicity, etc. [27]. In this model, the animals were all in a sedated and stable state compared to a cohort of heterogenic ICU patients, in whom direct aspiration might not have been detected. Our results suggest that direct aspiration of drugs could be an unstudied factor contributing to variations in antibiotic concentrations in patients receiving CRRT. This study further marks the need to monitor drug concentration in critically ill patients, if possible [28].

The clinical importance of our results certainly depends on the infused drug. Many drugs in intensive care (vasopressors, insulin and sedatives) are infused to a clinically given target. Direct aspiration could lead to an insufficient drug response and possibly to the introduction of a second line of drug, for example, a high dose of noradrenalin and, thereby, introduction of vasopressin. This might expose the patient to unnecessary risks and drug burden. It is also possible that the relationship between the two catheter tips is even more important when giving a bolus dose of a drug, because of enhanced direct aspiration, due to a higher infusion rate.

The limitations of our study are first that it is performed in healthy animals in an experimental setting. However, this is also a strength, since the results might have been clouded in a heterogenic ICU setting as mentioned above.

Another limitation is the fact that ICU patients, receiving CRRT, often suffer from renal impairment which can affect clearance of drugs. In this model, the piglets had no previous renal insult, and therefore, primary outcome was dialysis clearance and not total clearance. If this difference has any implications when translating, this experiment to the ICU setting is not evidently clear.

Finally, another limitation is the short observation time at each measuring point which might have reduced the opportunity to discover a change in serum concentration. However, the primary endpoint was dialysis clearance and the model was constructed to achieve that endpoint.

To our knowledge, this is the first in vivo study to show that the position of CVC and CDC, in the same vessel, is important for drug clearance during CRRT. The clinical implications of these findings will be dependent on the infused drug.

## Conclusion

The distance between CVC and CDC tips can affect drug clearance during CRRT.

A cranial versus a caudal tip position of the CVC in relation to the tip of the CDC led to the highest dialysis clearance.

A clinical study on direct aspiration is necessary to further guide placement of multiple catheters in the same patient.

## Data Availability

The data set analysed in this study is available from the corresponding author upon reasonable request.

## Abbreviations

AB: Antibiotic
C: Clearance
CO: Cardiac output
CVC: Central venous catheter
CDC: Central dialysis catheter
CVVHD: Venovenous haemodialysis
CRRT: Continuous renal replacement therapy
ECMO: Extra corporeal membrane oxygenation
EJV: External jugular vein
GM: Gentamicin
Hb: Haemoglobin
HR: Heart rate
ICU: Intensive care unit
ICV: Inferior caval vein
IQR: Interquartile range
L/min: Litres per minute
MAP: Mean arterial pressure
mg/ml: Milligram per millilitre
mmHg: Millimetres of Mercury
mol/L: Millimoles per litre
p: Pressure
P: Plasma
p_a_CO_2_: Arterial partial pressure of carbon dioxide
PEEP: Peak end expiratory pressure
RRT: Renal replacement therapy
S: Serum
SD: Standard deviation
SCV: Superior caval vein
VM: Vancomycin
